# The Ordered Extension of Pseudopodia by Amoeboid Cells in the Absence of External Cues

**DOI:** 10.1371/journal.pone.0005253

**Published:** 2009-04-22

**Authors:** Leonard Bosgraaf, Peter J. M. Van Haastert

**Affiliations:** Department of Cell Biochemistry, University of Groningen, Haren, The Netherlands; University of Birmingham, United Kingdom

## Abstract

Eukaryotic cells extend pseudopodia for movement. In the absence of external cues, cells move in random directions, but with a strong element of persistence that keeps them moving in the same direction Persistence allows cells to disperse over larger areas and is instrumental to enter new environments where spatial cues can lead the cell. Here we explore cell movement by analyzing the direction, size and timing of ∼2000 pseudopodia that are extended by *Dictyostelium* cells. The results show that pseudpopod are extended perpendicular to the surface curvature at the place where they emerge. The location of new pseudopods is not random but highly ordered. Two types of pseudopodia may be formed: frequent splitting of an existing pseudopod, or the occasional extension of a *de novo* pseudopod at regions devoid of recent pseudopod activity. Split-pseudopodia are extended at ∼60 degrees relative to the previous pseudopod, mostly as alternating Right/Left/Right steps leading to relatively straight zigzag runs. *De novo* pseudopodia are extended in nearly random directions thereby interrupting the zigzag runs. Persistence of cell movement is based on the ratio of split versus de novo pseudopodia. We identify PLA2 and cGMP signaling pathways that modulate this ratio of splitting and *de novo* pseudopodia, and thereby regulate the dispersal of cells. The observed ordered extension of pseudopodia in the absence of external cues provides a fundamental insight into the coordinated movement of cells, and might form the basis for movement that is directed by internal or external cues.

## Introduction

The movement of amoeboid cells is mediated by actin-filled protrusions of the cell surface, pseudopodia [1]. It is often thought that in the absence of external cues, cells extend pseudopodia in random directions, and that spatial cues such as chemoattractants induce a bias in the size or direction of the pseudopodia [2]. As early as 1953, however, it was shown that in the absence of external cues, cells exhibit a so-called correlated random walk [3], an observation that has been reproduced for nearly all moving cells [4]–[7]. Correlated means that a cell is more likely to move in a direction similar to its previous direction of movement. This tendency to move in the same direction is called persistence, and the duration of the correlation is the persistence time.

What is the function of persistence versus random movement and how can cells move in a persistent manner? Cells with very short persistence times approach a random walk with many turns and consequently move chaotically in a small area. In contrast, cells with strong persistence make few turns, move for prolonged periods of time in the same direction, and thereby penetrate the environment. This suggests that persistence may have a major impact on how cells colonize a new environment, such as during food seeking, morphogenesis and metastasis. Chemotaxis may represent another field of cell biology where persistence could be critical. It is thought that during chemotaxis positional cues induce a bias of pseudopod extension, by which cells move on average more often in the direction of the chemoattractant gradient than in other directions. Cells moving without persistence need a chemotaxis bias for each new pseudopod, while cells moving persistently will accumulate directional accuracy at each subsequent pseudopod.

The mechanism of persistent cell movement is likely to be founded in how cells extend series of pseudopodia [8]. Previous studies have investigated persistent cell movement by tracking the centroid of the cell [3]–[5], [9], [10]. Other studies have analyzed the shape of cells using autocorrelation to reveal ordered patters of shape changes that are masked by noise [11]. The obtained results have been interpreted in terms of basic elements of cell movement, such as steps and turns, or ordered protrusions. We have chosen for an opposite strategy on the assumption that the extension of a pseudopod is the basic element for cell movement, and that shape changes and cell trajectories are the consequence of the pattern of pseudopod extension. Therefore, we have developed a pseudopod tracking tool that identifies the position and time of the start and end of pseudopod growth [12]. Each pseudopod is thereby described as a vector with length, direction and timing. In the present study we have explored pseudopod extension in the absence of external cues. We collected vector data for ∼2000 pseudopodia that are extended by starved *Dictyostelium* cells in buffer. These data were used to characterize a highly ordered pattern of pseudopod extension with respect to the angle between subsequent pseudopodia and the position at the cell surface where pseudopodia emerge. We discuss the consequences of ordered pseudopod extension for the trajectories of cells, and for the mechanisms that cells may use to respond to external cues such as chemoattractants.

## Results

**Figure 1:**
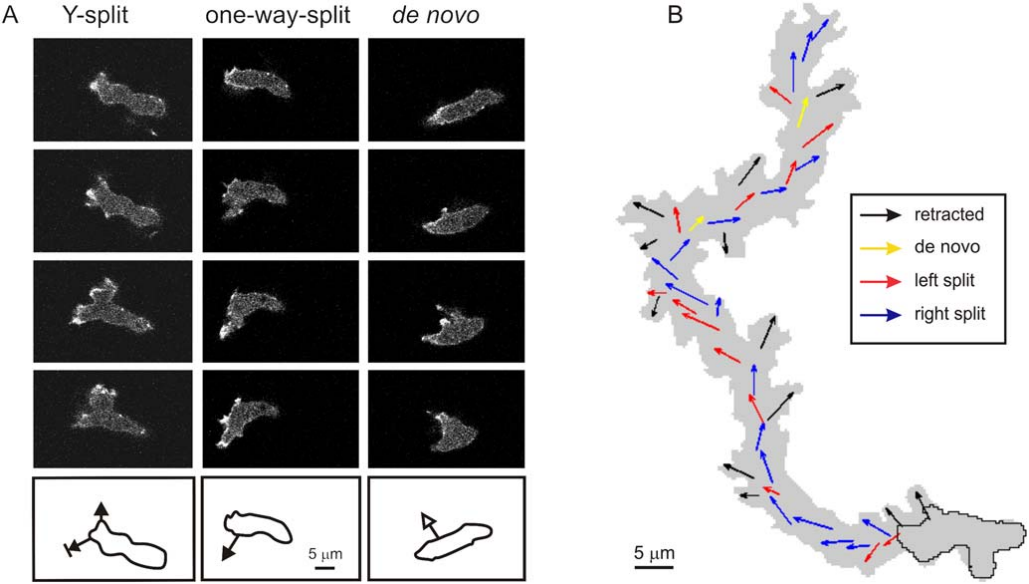
Pseudopod extensions. A. *Dictyostelium* cells extend two types of pseudopodia, split and *de novo*. Left: Y-shape split. An existing pseudopod splits in two that are both protruded. Finally, the left pseudopod is retracted while the right pseudopod survives. Middle: One-way split. A protrusion is formed from the basis of an existing pseudopod; the cytoplasm flows into this new pseudopod, but not in the existing pseudopod. Right: *De novo* pseudopod. A slender protrusion is formed at an area of the cell that did not exhibit pseudopod activity in the previous two minutes. The cytoplasm flows into this new pseudopod. The images are at 8 s interval. The diagrams below the confocal images depict the pseudopod as arrow with the contour of the cell in the upper image. B. Track of a cell moving during 14 minutes in buffer (see movie S1 in supplemental information). The grey area indicates the contour of the cell during this movement. The arrows show the pseudopodia. As presented in figure 3, split pseudopodia are often alternating right/left leading to relatively straight path, while *de novo* pseudopodia are in random directions causing a change of direction.

*Dictyostelium* cells, like neutrophils and many other amoeboid cells, can extend two types of pseudopodia [13]. The first arises from splitting of an existing pseudopod and is often the predominant type of newly formed protrusions. The cells may also extend pseudopodia from areas of the cell not previously active, which we describe as *de novo* pseudopodia (often referred to as “lateral pseudopodia” because they often appear at the side and in the rear of the cell). The two modes of pseudopod initiation are shown in Fig. 1A. During pseudopod splitting, first ruffles appear at the base of an existing pseudopod that subsequently develop into a major pseudopod. In the minority of cases (∼10%) pseudopod splitting leads to two equivalent extensions (Y-shape), one of which eventually retracts while the other remains. The majority of cases are dead-end splits: the cell body flows into the newly extending pseudopod, while the old pseudopod is not extended but merges with the cell body. The *de nov*o pseudopodia often start as slender extensions that become wider as they incorporate the cell body (Fig. 1A).

### Pseudopod tracking

The aim of this study is to deduce how cells extend pseudopodia and to use this knowledge to understand the tracks of moving cells. Therefore, we determined the space-time co-ordinates of the tip of the pseudopod when it started and stopped its extension, respectively. Initially, movies were analyzed manually with support of a pseudopod tracking program. The investigator indicates the start and end position of an extending pseudopod, and the program places a hard-copy arrow on the relevant images of the movie, and exports the space-time co-ordinates of start and end point (see methods). Pseudopodia were subsequently annotated by the investigator either as formed by splitting or *de novo*, maintained or lost, and extended to the right or left relative to the direction of the previous pseudopod.

After analyzing several thousand pseudopodia, we learned to describe pseudopod extension, and to program a fully automated pseudopod-tracking algorithm, Quimp3 [12]. The method is based on the observation that a pseudopod has a convex curvature, and that pseudopod extension starts and stops rather abruptly. The first algorithm uses an active contour [14] to describe the outline of a cell as ∼150 bar-coded nodes. By comparing the position of the nodes in subsequent frames, the algorithm identifies extending and retracting regions of the cell. A second algorithm identifies an active pseudopod as an extending area with convex nodes. The algorithm identifies the tip of the pseudopod as the node in the center of the convex area, and exports the x,y,t coordinates of this tip node at the start and end of the growth period. The third algorithm annotates the pseudopodia as de novo or splitting, maintained or lost, and right or left relative to the previous pseudopod. The output file of the program contains many quantitative details that have been used to calculate properties of pseudopodia such as average size, growth time, and interval for wild type and mutant cells (see supplemental table S1 for primary data).

Fig. 1B presents the cell track with extended pseudopodia of a typical 5 h starved cell moving in buffer, obtained by the fully automated method, and reveals that the path is composed of mainly pseudopod splitting and occasionally *de novo* pseudopodia. The sequence of splitting pseudopodia leads to a relatively straight persistent cell-track, while a *de novo* pseudopod may induce a change of direction.

### Time of pseudopod extension

**Figure 2:**
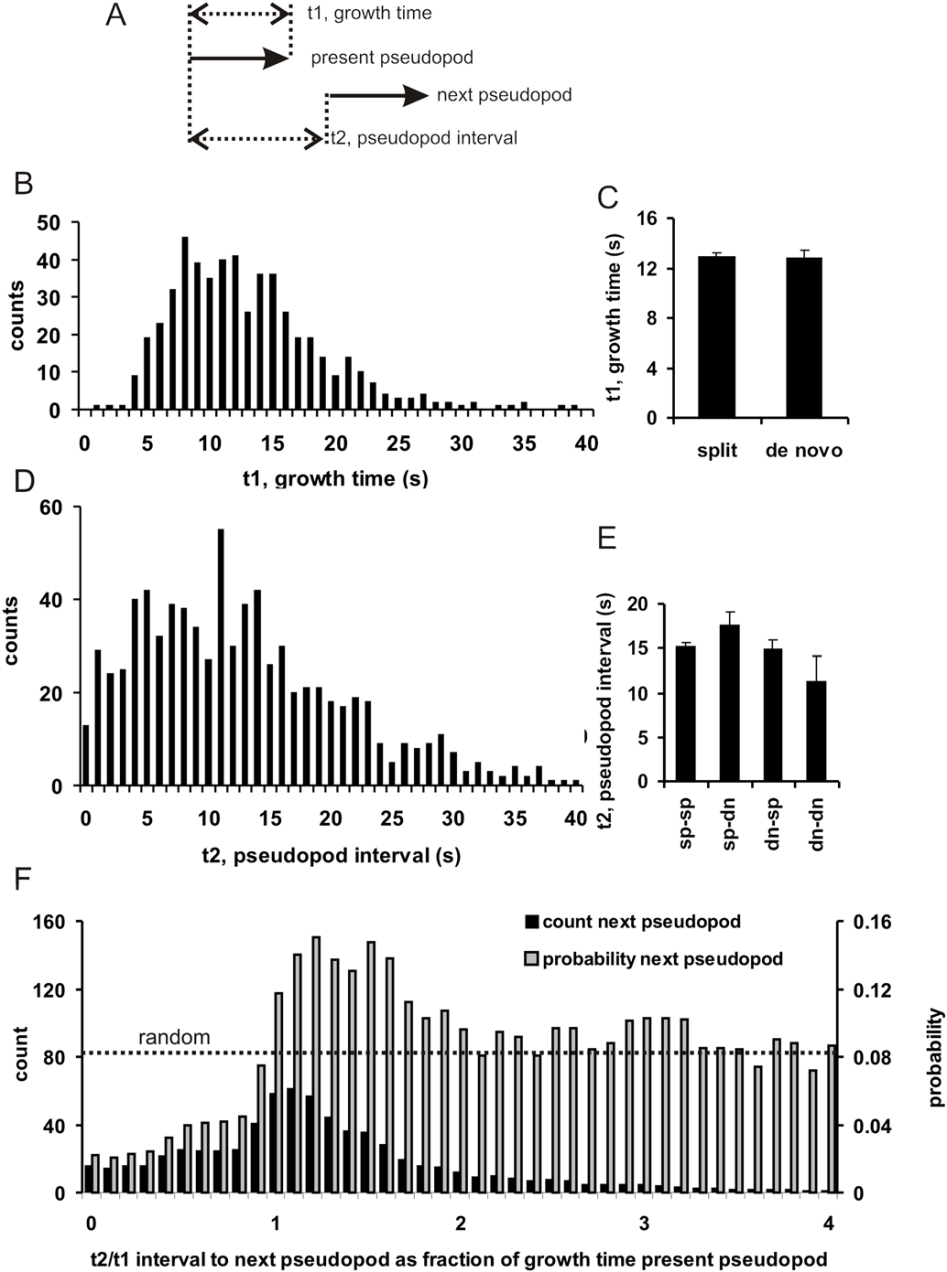
Timing of pseudopod formation. A. Schematic of the experiment. The two arrows indicate the start and finish of two pseudopodia. Panels B and D are probability frequency distributions of growth time and pseudopod interval, respectively, determined for 896 pseudopodia. Split and de novo pseudopodia have similar growth time (C) and pseudopod interval (E; sp = split, dn = de novo); data are means and SEM. Panel F presents the time point at which a new pseudopod starts during or after growth of the present pseudopod (1 means that the new pseudopod starts at the moment that the present pseudopod stops growth). Data are binned in 0.1 intervals. The grey bars indicate the probability that the next pseudopod will start during the indicated interval (see text for equation). The probability for a random start is given by (bin interval)x(mean t2)/(mean t1) = 0.1×12.9/15.7 = 0.082. The results show that the start of a new pseudopod is inhibited during growth of the present pseudopod, but is transiently activated immediately after the stop of the present pseudopod.

We determined two temporal components of pseudopod extensions: the pseudopod growth period *t1* and the interval between the extensions of two pseudopodia *t2* (see Fig. 2A). The average growth period *t1* is 12.8 s with rather large variation (SD = 5.4 s, n = 896 pseudopodia; Fig. 2B). Previously we observed that the pseudopod tip changes within one second from a low basal speed before growth to a high constant speed during growth [12]. Together with the present data this suggests that there is substantial stochastic variation in the period of pseudopod growth, but when growth comes to an end, it stops suddenly within 1 s. The pseudopod interval is the time period between the start times of two subsequent pseudopodia. The average pseudopod interval is 15.3 s, about 3 s larger than the pseudopod growth time. Thus on average a new pseudopod starts ∼3 s after the previous pseudopod stops growing. However, there is a high degree of variation in pseudopod interval (Fig. 2D), and new pseudopodia frequently emerge while the previous pseudopod is still growing. We determined when a new pseudopod begins during or after the growth of the present pseudopod (presented as t2/t1; Fig. 2F). Although some new pseudopodia emerge just after the previous pseudopod has started (t2/t1 close to 0) or long after the previous pseudopod has stopped (t2/t1>2), most new pseudopodia start slightly after the present pseudopod has stopped (t2/t1 just above 1). We calculated for these 724 pseudopodia the probability *P(i)* that a cell extends the new pseudopod in the time interval (*i*), which is the number of cases *p_i_* that cells extends a pseudopod in that interval divided by the number of cases that cells have not yet extended a new pseudopod, i.e. *P(i) = p_i_/(1−Σp_i−1_)*. The results show that, compared to the random extension of pseudopodia, the probability to extend a new pseudopod is inhibited by 60 to 70% during the growth period of the present pseudopod (t2/t1<1). In contrast, pseudopod extension is activated about 60% immediately after the present pseudopod stops. This activation is transient, because the probability declines to values expected for random extension for the rare events of very late new pseudopodia.

The aforementioned data were obtained for all pseudopodia. We determined some properties of split and *de novo* pseudopodia, separately. The growth period is not significantly different between split and de novo pseudopodia (Fig. 2C). Two subsequent pseudopodia can be split-split, split-novo, novo-split and the rare novo-novo. The interval between two subsequent pseudopodia is also not significantly different between these four cases (Fig. 2E). Pseudopodia have a length between about 2 and 10 µm (see supplemental figure S1). Split and de novo pseudopodia have approximately the same length distribution, with an average of 5.9+/−2.3 µm for split and 5.3+/−1.8 µm for de novo (mean and SD, n = 530 split and 112 de novo).

### Angle of pseudopod extension

**Figure 3:**
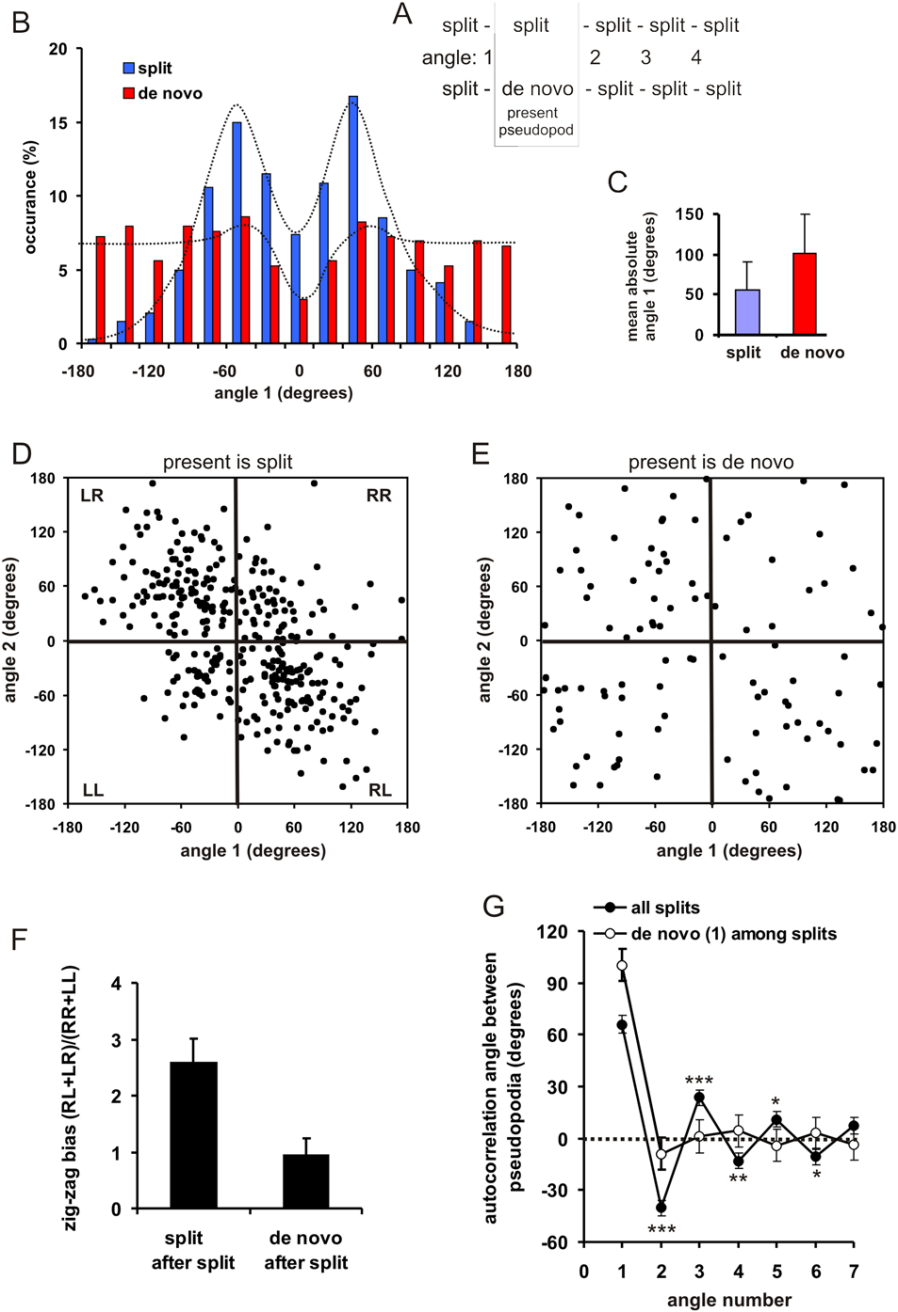
Direction of pseudopod extension. A. Schematic of the experiment. Series of split pseudopodia were analyzed in which the present pseudopod is either a split or a de novo pseudopod. Angle 1 is the angle between the present pseudopod and the previous pseudopod. B and C, probability frequency distribution of angle 1 showing bimodal distribution for split pseudopodia with mean of about 55 degrees, and a broad distribution for de novo pseudopodia with a mean of 100 degrees. D–F, presents the angle of two subsequent pseudopodia, and shows that split-split exhibit a bias towards alternating steps (RL and LR) versus consecutive hops (RR and LL). De novo pseudopodia do not exhibit a right/left bias. The data of panel F are the means and SD of 16 cells. Panel G presents the autocorrelation of angle 1 with the angles of subsequent pseudopodia; the error bars indicate the SEM with n = 196 for split and n = 190 for de novo pseudopodia. The angle of a split pseudopod is negatively correlated with the angle of the following pseudopodia during 6 splits at a significance ***P<0.001; **P<0.01; *P<0.05. The angle of a de novo pseudopod is not correlated with the subsequent split pseudopodia.

The path of a cell is determined to a large extend by the angle between subsequent pseudopodia. We selected all longer series of split pseudopodia in which the second pseudopod is either a split or a de novo pseudopod (Fig. 3A), and determined the angles *φ* between two subsequent pseudopodia. The angle between two split-split pseudopodia is bimodally distributed with peaks of about 55 degrees to the right or left relative to the previous pseudopod (Fig. 3B and 3C). In contrast, a de novo pseudopod is extended with equal probability in nearly all possible directions, except in the direction of the previous pseudopod; the mean angle is 101+/−49 degrees, slightly larger than random (90 degrees).

A pseudopod can extend to the right (R, positive angle) or to the left (L, negative angle) relative to the previous pseudopod, and therefore two subsequent split pseudopodia may be alternating (RL or LR, denoted as a step) or consecutive (RR or LL, denoted as a hop). Are steps equally probable as hops, or do cells more frequently make alternating RLR steps leading to persistent movement? To answer this question we plotted the angle between first and second pseudopod against the angle between second and third pseudopod. When all three pseudopodia are split (Fig. 3D), the two angles are clustered in the RL and LR quadrants; the alternating RL+LR steps occur about 3 times more often that the consecutive RR+LL hops (Fig. 3F). In contrast, when the second pseudopod is a de novo, the angles are homogeneously distributed among the four quadrants, and RL+LR steps are equally probable as RR+LL hops (Fig. 3E).

To investigate how long the alternating RLR bias persists we selected N = 196 series of at least 8 split pseudopodia, and calculated the autocorrelation *C_θθ_(i)* between the first angle *θ(1)* with all subsequent angles *θ(i)* using the equation 
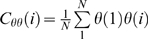
. The autocorrelation of the angle of splitting pseudopodia is clearly alternating positive and negative; the amplitude declines at each subsequent pseudopod, but is still significantly different from random after 6 split pseudopodia (Fig. 3G). In contrast, a de novo pseudopod in a series of splitting pseudopodia immediately reduces the autocorrelation to insignificant values. In summary, the data of figure 3 reveal that splitting pseudopodia are extended at a relatively small angle of 55 degrees with a strong alternating right/left bias, thereby providing persistence of movement. In contrast, de novo pseudopodia are extended in nearly random direction, have no right/left bias, and interrupt the right/left bias of split pseudopodia. Thus, de novo pseudopodia randomize the direction of movement.

### Pseudopodia are extended perpendicular to the cell surface

**Figure 4:**
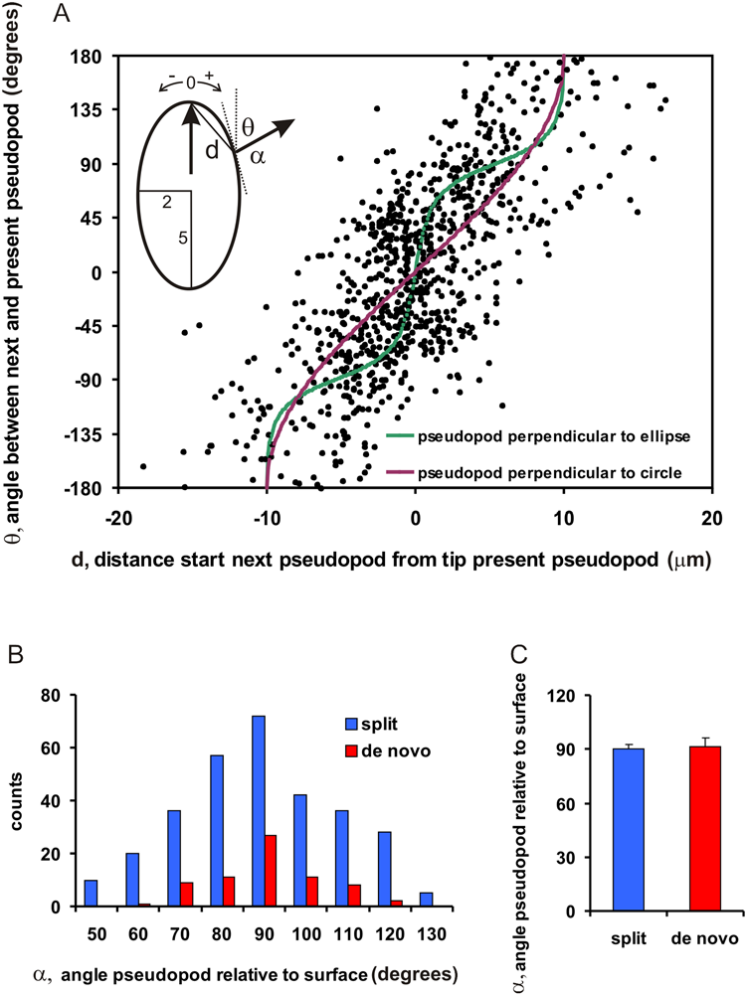
Pseudopodia are extended perpendicular to cell curvature. A. The angle *θ* between the present pseudopod and the previous pseudopod is plotted versus the distance d between the start of the present pseudopod and the tip of the previous pseudopod, as indicated in the inset. The lines represent the theoretical curves for pseudopodia that are extended perpendicular to a circle (purple with radius 5 µm) or an ellipsoid (green ). B. The angle *α* was determined that is formed by the direction of the pseudopod and the tangent to the cell boundary at the position of pseudopod emergence. Panel B shows the frequency distribution, while panel C presents the means and SEM of 306 split and 69 de novo pseudopodia. The angle *α* is statistically not significantly different from 90 degrees.

Figure 4A reveals that the angle *θ* between present and next pseudopod increases when the next pseudopod emerges further away from the present pseudopod. This observation could be explained by the simple hypothesis that pseudopodia are extended perpendicular to the local surface curvature of the cell, because the geometry of a circle or ellipsoid predicts that perpendicular pseudopodia emerging at a longer distance will have a larger angle *θ*. Figure 4A shows the calculated curves for the angle *θ* as a function of the distance between two points on a circle with radius of ∼5 µm (purple line) and an ellipsoid (green line) with long and short axis of ∼5 and ∼2 µm [15]. The observed distance dependency of the angle between pseudopodia is consistent with pseudopod extension perpendicular to the surface of a cell.

The hypothesis of perpendicular pseudopodia would also explain the observed difference between the angle of split and de novo pseudopodia: Split pseudopodia are extended nearby (average ∼4 µm) and thereby at a small angle (55 degrees), while de novo pseudopodia are extended further away (∼8 µm) and thereby at a larger angle (100 degrees). To test the hypothesis we measured the angle *α* between the pseudopod and the local cell curvature (more precisely, the angle with the tangent to the contour at the node where the pseudopod started). The observed angle between pseudopod and local membrane curvature is 90.1+/−17 degrees (mean and SD, n = 220), and is not different between split and de novo pseudopodia (Fig. 4B and 4C). Visual inspection confirms that no pseudopodia are extended at an angle <40 degrees.

### Split and de novo pseudopodia in signaling mutants

**Figure 5:**
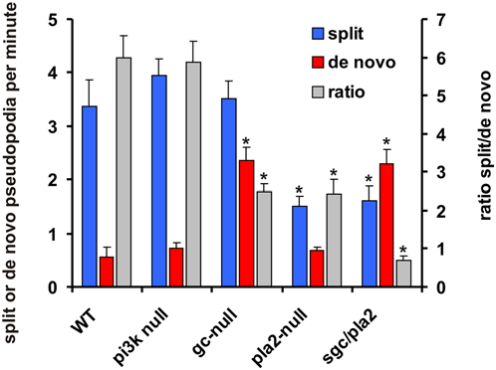
Pseudopod behavior of signaling mutants. Wild-type cells (WT), *pi3k1/2*-null, *gc*-null, *pla2*-null and *sgc/pla2*-null cells were starved for 5 hours. The frequency of split and *de novo* pseudopodia is presented, as well as the ratio of split/de novo pseudopodia. The data shown are the means and SEM of 7 to 12 cells (*, significantly different from WT at P<0.01).

Wild type cells extend approximately 3.5 split pseudopodia and 0.7 de novo pseudopodia per minute (Fig. 5). The split/de novo ratio is *a* = 6.0+/−1.0; i.e. on average a de novo pseudopod is followed by 6 split pseudopodia. Recently it was demonstrated that movement of *Dictyostelium* cells towards the chemoattractant cAMP is mediated by at least three signaling enzymes, PI3-kinase, PLA2 and a soluble guanylyl cyclase sGC [16]–[18]. We measured pseudopod behavior in 5 h starved mutant cells defective in these signaling pathways. The results show that *pi3k*-null cells that lack two important *pikA* and *pikB* genes exhibit similar pseudopod splitting and de *novo* pseudopod formation as wild type cells (Fig. 5). Previous experiments have shown that suppression of *de novo* pseudopodia depends on a cGMP-mediated signaling pathway leading to myosin filament formation at the sides and in the rear of the cell [19]. Accordingly, a *gc*-null mutant that lacks both *gca* and *sgc* genes that together encode for all guanylyl cyclase activity extends ∼3 times more *de novo* pseudopodia; at the observed unaltered frequency of pseudopod splitting this leads to a strong reduction of the average length of the series of split pseudopodia from *a* = 6 in wild type to *a* = 2.5 in *gc*-null cells. Cells lacking the *pla2A* gene also exhibit a small value for split/de novo ratio (*a* = 2.4), but this is due to a strong reduction of pseudopod splitting instead of enhanced *de novo* pseudopodia formation. The double *sgc/pla2*-null cells lacking both guanylyl cyclase and PLA2 activity demonstrate both defects of enhanced *de novo* pseudopodia and reduced pseudopod splitting. The total pseudopod frequency is similar to that of wild type cells, but with a split/de novo ratio of *a* = 0.7 *sgc/pla2*-null cells mainly extend de novo pseudopodia.

### Consequence of de novo and split pseudopodia for cell movement

**Figure 6:**
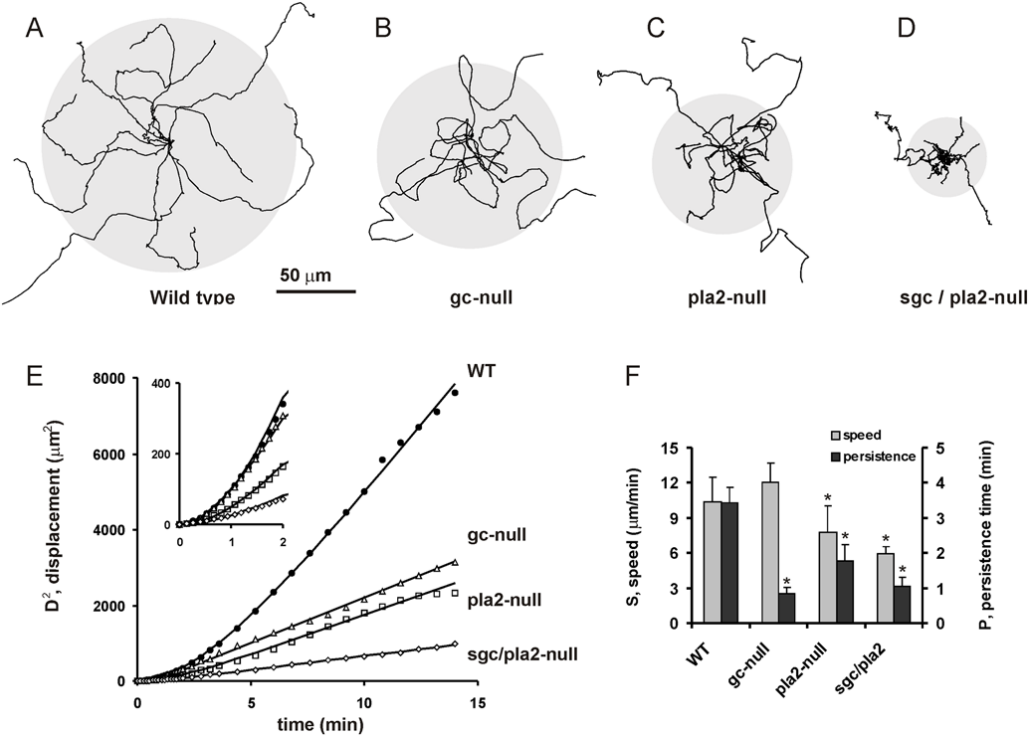
Dispersion of wild type and mutant cells. Movies of 5 h starved wild type and mutant cells were recorded in 2.5 mM caffeine to inhibit cAMP signaling. Long cell tracks of at least 20 minutes were analyzed. Panels A–D show the tracks of 10 cells during 15 min; the grey circle indicates the average dispersal. Panel E shows the dispersal during time interval t; the symbols indicate the measured data, and the curves are the fit of the data to the equation of persistent movement (see text). The fitted parameters for speed (S) and persistence time (P) are presented in panel F; the error bars represent the 95% confidence limit (*, significantly different from WT at P<0.01).

The aim of this study was to unravel how cells extend pseudopodia, and from there to understand how cells move in longer trajectories. Amoeboid movement is a typical persistent random walk [4], [5] following the equation 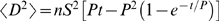, where *D* is the displacement, *n* is the dimensions of dispersal (here two dimensions), *S* is speed and *P* is persistence time. We measured the displacement of wild type cells during 15 minutes (see Fig. 6A), and fitted the observed mean square displacement to this equation, yielding a speed of *S* = 10.4+/−2.1 µm/min and a persistence time of *P* = 3.4+/−0.5 min (mean and 95% confidence; Fig. 6E,F). In the context of the present study on pseudopodia, the persistence time is the duration that maintained pseudopodia are extended in the same direction, and is given by *P = (1+a)/M*, where *a* denotes the number of splits followed by one de novo pseudopod (observed *a* = 6.0+/−1.0) and M denotes the frequency of maintained pseudopodia (observed M = 2.05+/−0.15 min^−1^; see table S2). These data result in a calculated persistence time for pseudopod extension of *P* = 3.41+/−0.62 min, identical to the observed persistence time of cell tracks.

We recorded the trajectories of mutant cells with altered ratios of de novo/splitting pseudopodia. The gc-null cells, with enhanced de novo pseudopod extension, disperse during 15 min over a significantly smaller area than wild type cells (Fig. 6B). Cells move for a shorter period in the same direction and make more turns. From the persistence plot we obtained a slightly larger speed of *S* = 12.0+/−1.7 µm/min that is statistically not different from the speed of wild type cells, and a persistence time *P* = 0.83+/−0.18 min that is considerably shorter than the persistence time of wild type cells. The results show that gc-null cells extend more pseudopodia per minute than wild type cells, but a large fraction of these pseudopodia are de novo in a new random direction. The dispersal of pla2-null cells with reduced splitting is also smaller than wild type cells (Fig. 6C). Here the analysis reveals a reduced speed (*S* = 7.7+/−2.3 µm/min) and a reduced persistence time (*P* = 1.7+/−0.5 min). These pla2-null cells extend significantly less split pseudopodia than wild type cells, explaining both the reduced speed and persistence. The double *sgc/pla2* mutant disperses very poorly (Fig. 6D). The speed has reduced nearly 50% compared to wild type cells (*S* = 5.9+/−0.6 µm/min). Importantly, pseudopod activity, defined as the product of pseudopod size and frequency, is similar in *sgc/pla2*-null cells (20.8+/−1.7 µm/min) and wild type cells (21.6+/−2.0 µm/min), indicating that *sgc/pla2*-null mutant cells actively extend pseudopodia. However, the persistence time *sgc/pla2*-null mutant cells is very short (*P* = 1.0+/−0.2 min), in accordance with the observation that nearly all protrusions are *de novo*, and cells constantly move in a new direction, i.e. cells wiggle as in Brownian motion and do not disperse effectively.

## Discussion

In this study we analyzed the movement of *Dictyostelium* cells from the perspective of pseudopod extension. We designed an algorithm that can automatically track pseudopodia. The method is based on the active contour program, Quimp, that describes the outline of a cell as a polygon of nodes [20]. By comparing the position of nodes in space and time, each node contains information on the local speed and curvature of the boundary [14]. The pseudopod algorithm uses local curvature and rapid area change to identify extending pseudopodia. We observed that before pseudopod initiation, the future tip of the pseudopod moves at a low rate, but reaches a maximum within one second after pseudopod emergence, and stays at that rate during the subsequent growth period.

### Geometry of cells and direction of pseudopodia

Pseudopodia are extended approximately perpendicular to the membrane (90+/−17 degrees). This observation may have a simple explanation. When an F-actin filled protrusion starts extension, it will induce a tension of the membrane. This counterforce is asymmetric when the pseudopod does not start perpendicular to the membrane. Unless the emerging pseudopod is mechanically locked intracellular, these asymmetric counterforces will correct the direction of the extending protrusion till the direction is perpendicular to the membrane. The notion that pseudopodia are extended perpendicular to the cell surface implies that the direction of the pseudopod, and consequently the direction of cell movement, depends on the local curvature of the cell boundary at the position where the pseudopod emerges. Two nearby pseudopodia that emerge from a smooth surface are extended in a similar direction, whereas the direction is very different when the surface is very irregular or the pseudopodia emerge at a large distance. We conclude that the geometry of the cell and the position of pseudopod induction dominate the direction of cell movement.

### Coordinated extension of pseudopodia

In the absence of external cues the extension of pseudopodia is not random, as deduced from the time, position and direction of the extension of the next pseudopod relative to the present pseudopod. First, the probability to extend a new pseudopod is small when the present pseudopod is still extending, but increases strongly during a short period after the present pseudopod has stopped (Fig. 2F). Second, the place where a new pseudopod emerges is not random. *Dictyostelium* cells, as many other eukaryotes, may extend two types of pseudopodia, splitting of an existing pseudopod, or de novo from an area that is devoid of recent pseudopod activity. Two nearby points on a sphere have tangents with a small difference in slopes, and two lines that are perpendicular to these tangents cross at a small angle. Thus, geometry predicts that split pseudopodia are extended on average at a small angle. In contrast, de novo pseudopodia are extended at a long distance from the present pseudopod, and therefore at a large angle. Third, split pseudopodia are extended with a right/left bias, preferentially as alternating steps relative to the consecutive hops. Importantly, the left/right bias is not observed for de novo pseudopodia.

**Figure 7:**
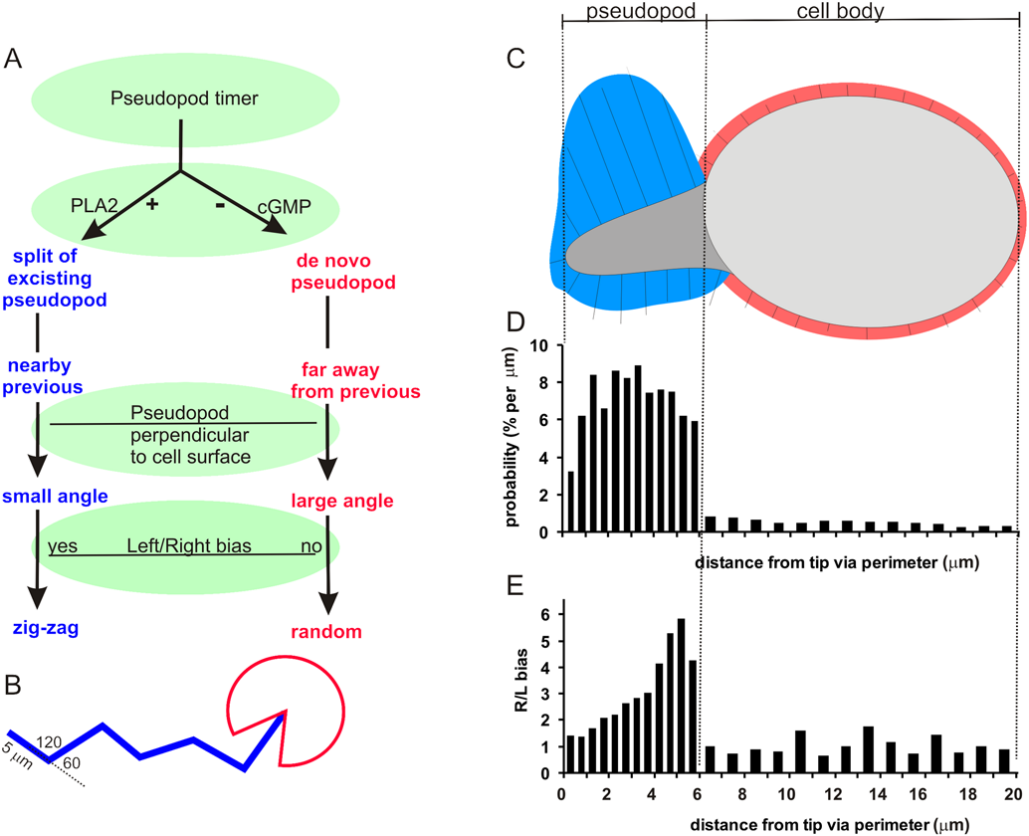
Schematics of pseudopod extension in *Dictyostelium*. A. A pseudopod timer initiates the extension of a new pseudopod every ∼15 seconds. Depending on the activity of PLA2 and guanylyl cyclase, this pseudopod is formed by splitting of an existing pseudopod or formed de novo on the cell body. The combination of pseudopodia being extended perpendicular to the cell surface, with distance and Left/Right bias is responsible for the relatively straight zig-zag trajectory of split pseudopodia and the random direction of a de novo pseudopod as indicated in panel B. Panel C shows a ‘model’ cell that has extended a pseudopod to the left, and is going to extend a new pseudopod. The model cell was constructed by separately averaging 30 pseudopodia and cell bodies (see discussion). The model pseudopod has a length of 5 µm, which is 6 µm via the perimeter; the cell body has a length of ∼11 µm, which is 14 µm via the perimeter. The length of the lines is proportional to the probability of pseudopod extension per micrometer. D. The probability that a new pseudopod is extended at different distances from the tip of this model cell was determined, and is expressed as % per µm perimeter of the model cell. E. The probability that the new pseudopod is extended to the right or left was calculated for the pseudopodia that emerged at different distances from the tip of the left-going pseudopod. The line segments that are drawn perpendicular to the model cell indicate the probability that a pseudopod is extended at that position. This pseudopod projection map suggests that amoeboid movement in the absence of external cues is orchestrated by mechanisms that inhibit pseudopodia in the cell body, and promote pseudopodia by splitting of the present pseudopod, but not at the tip, and not at the left side of a left-going pseudopod.

We were concerned about the possibility that the distinction between split and de novo pseudopodia is only based on the place where a pseudopod is formed, and is not related to fundamental biological differences between pseudopodia. The right/left bias may be an intrinsic property of split pseudopodia, as we propose. Alternatively, the right/left bias may be related to the polarity of the cell: it is strong in the front of the cell (where new pseudopodia emerge predominantly by splitting) and declines towards the rear of the cell (where pseudopodia appear de novo). Therefore, we determined the right/left bias for newly emerging split and de novo pseudopodia as function of the distance from the tip of the present pseudopod (see Figure S2 in supplemental information, and summarized in Fig. 7D). The results clearly support our proposal: the right/left bias of a new pseudopod that is extended at the surface of a present pseudopod (split) actually becomes stronger at larger distances from the tip, while the right/left ratio of a new pseudopod that is extended from the cell body (de novo) remains unbiased at any distance. Although we can not exclude the contribution of a polarity system which is stable on a longer time scale than an individual pseudopod, the results strongly suggest that the right/left bias is an intrinsic property of splitting pseudopodia.

### Pseudopod extension as basis for the trajectory of cell movement

Trajectories of moving cells are often presented as the position of the centroid of the cell in time; the centroid is the geometric center of the cell. Pseudopod extension is the basis for the trajectory of the cell, but the connection between pseudopod extension and centroid is not simple. Assume a stationary spherical cell (radius 5 µm, surface area of 2D image ∼78 µm^2^) that extends a pseudopod of 5 µm long and 3 µm wide at its base (surface area ∼7.5 µm^2^). When the cell body does not move, the extension of the pseudopod will lead to the movement of the centroid in the direction of the pseudopod by only ∼0.25 µm. When new pseudopodia are extended constantly in a new direction, the cell wiggles with small displacements; at the observed frequency of 4 pseudopodia per minute, the speed will be only ∼1 µm/min. In contrast, when all pseudopodia are extended in the same direction, the cell body must follow the pseudopod, resulting in a speed of maximally 20 µm/min. The actual speed is lower (∼10 µm/min), because only ∼60% of the pseudopodia of starved cells contribute to cell movement; they are identified by the pseudopod algorithm as “maintained”. The other ∼40% of the pseudopodia are “lost”; either they are retracted rapidly, or they are extended in a direction that is not followed by other pseudopodia and the cell body. Therefore, both the speed of the cell and the shape of the trajectory depend on the persistence of maintained pseudopodia to continue movement in a chosen direction.

The pioneering work of Potel and Mackay on cell trajectories of *Dictyostelium* movement revealed details on velocity and persistence time (4.9 min), but the time resolution of the movies (15 s/frame) did not preclude further details [4]. More recently, Takagi et al. performed a detailed statistical analysis of trajectories with high temporal resolution (1 s/frame). Two time scales in velocity autocorrelations were observed: 5–11 s, which was interpreted as the potential filopod (or pseudopod) formation dynamics, and 3.8 min that may be due to the persistence time of directional movement [10]. These data are fully consistent with our observations on pseudopod extension. Li et al. recorded cell tracks at a lower temporal resolution (10 s/frame), and subdivided the tracks in straight runs and turns [9]. Since the centroid of cells in trajectories exhibits moderate spatial and temporal resolution, the turn detection algorithm required a threshold for minimal time (40 s) and angle (∼15 degrees) between two turns. The average run time was about 75 s. Our data show that 90% of the pseudopod have extension time between 4 and 20 s, and pseudopod interval between 2 and 30 s, suggesting that the run/turn analysis of tracks does not have sufficient temporal resolution to deduce information on each individual pseudopodium. However, the results may be interpreted, knowing that each run may consist of 1, 2 or 3 pseudopodia. Li et al. observed that the turns were alternating right left, as we observed for pseudopod splitting. However, the quantitative data on run/turn of tracks are different from extension/split of pseudopodia: the average angle between turns is 78 degrees (pseudopod 55 degrees), the right/left memory is only 1 or perhaps 2 turns (pseudopod 5–6 splits), the left right bias is 2.1 (pseudopod 2.8), the average run length is ∼9 µm (pseudopod 5 µm), and time of run is ∼75 s (maintained pseudopod 29 s). We interpret the runs as the extension period of one or multiple pseudopodia and the turn as the first pseudopod splitting that occurs after the threshold of 40 s following the previous turn. The possible presence of multiple R/L splitting pseudopodia in one run explains the lower R/L bias of runs compared to splitting pseudopodia. The random turns by a de novo pseudopod were not observed in the tracks, because they are probably hidden in the noise due to the lower temporal and spatial resolution of the tracks compared to pseudopodia. With this interpretation, the trajectories are fully explained by the primary data on pseudopod extension.

We have concluded that starved *Dictyostelium* cells exhibit a persistent walk that is based on split pseudopodia *retaining* the direction of movement and de novo pseudopodia providing a *change* of direction. As a consequence, the persistence of the cell trajectory will depend on the proportion of split/de novo pseudopodia. Cells that extend only de novo pseudopodia will exhibit a random walk, while an increase of the proportion of splitting pseudopodia will lead to enhanced persistence. In starved wild type cells this split/de novo ratio is 6.0, which means that after a random de novo pseudopod on average six splitting pseudopods are extended in a similar direction. The frequency of de novo and splitting pseudopodia appears to be regulated by cGMP and PLA2 signaling. The sgc/pla2-double null cells have a low frequency of pseudopod splitting and high frequency of de novo pseudopodia. As a consequence, persistence is very low. Although cells extend pseudopodia at a similar frequency as wild type cells, mutant cells change direction so often that the centroid of the cell follows the pseudopodia only for a short distance, resulting in the observed low speed of the cells. In addition, due to these repeated changes of direction, the movement of mutant cells approaches a random walk with many short runs and repeated returns to previous positions.

### Projection map of pseudopodia

The quantitative data on pseudopod extension may be combined in a projection map that presents the probability of pseudopod extension at different positions of a “model” cell. This projection map is qualitative, because it assumes a 2-dimensional cell, and it eliminates the stochastic variation between cells. Nevertheless, a projection map can be helpful to discuss the potential mechanisms that underline the coordinated extension of pseudopodia. The junctions between cell body and pseudopod are very often concave areas, which were used to subdivide the cell in the leading pseudopod and the cell body. Subsequently we averaged separately the leading pseudopodia and the cell bodies of 30 cells that just completed a split pseudopod to the left. This results in a “model” cell that is composed of a nearly spherical cell body with radius ∼5 µm and a blunt-tapered pseudopod of ∼5 µm long and ∼3 µm wide at its base (Fig. 7C). The perimeter of the model cell is ∼20 µm from tip of the pseudopod to rear of the cell, of which ∼6 µm comprises the leading pseudopod and ∼14 µm the cell body. We expressed the x-coordinate of the start position of each new pseudopod as % distance from tip to base of the present pseudopod, or from front to rear of the cell body. Next, the data of 620 pseudopodia were binned in length units of the model pseudopod (100% is 6 µm) and cell body (100% is 14 µm), and presented as the probability of pseudopod extension per µm of surface (Fig. 7D). The total perimeter of the model cell is 40 µm, yielding a random probability of 2.5%/µm. The results show that ∼15% of the pseudopodia are formed de novo from the cell body with approximately uniform distribution over the cell body, which has a perimeter of ∼28 µm, resulting in the low probability of 0.5%/µm. With 85% of the new pseudopodia extended by splitting of the old pseudopod with a length of 12 µm, the average probability is much higher (7%/µm), but appears to be not uniform. First, the probability to extend a new pseudopod in the vicinity of the tip of the present pseudopod is low (∼4%/µm). Secondly, the probability to extend a pseudopod to the right is much higher than to the left. Figure 7C presents pseudopodia as line segments perpendicular to the surface of the model cell; the length of the line segments indicates the probability of pseudopod extension per µm of surface.

### Pseudopod extension by an excitable medium of activators and inhibitors

The qualitative projection map of pseudopodia on a model cell may be used to discuss potential activators and inhibitors of pseudopod extension. The term activators is used here in a broad sense, not only for molecules that actively induce a pseudopod, but also excitability of the medium to induce a new pseudopod, or the availability of essential cytoskeletal components. We may try to deduce some properties of these hypothetical activators and inhibitors that regulate a pseudopod timer, the selection of split versus de novo pseudopod, and the position of a split pseudopod.

It is conceivable that the start and finish of pseudopod extension is regulated by complex mixtures of activators and inhibitors that together form a pseudopod timer to induce on average one pseudopod every 15 seconds. We observed that induction of a new pseudopod is suppressed by the present growing pseudopod (Fig. 2F), and this suppression is not stronger or weaker when the next pseudopod is extended nearby or far away from the present pseudopod (data not shown). This may suggest that the extending pseudopod gives rise to a global inhibitor of pseudopod initiation. Shortly after a present pseudopod stops growing, a high incidence of the induction of a new pseudopod is observed during 6–10 seconds. Also here the induction of a new pseudopod is not faster or slower when the next pseudopod is extended nearby or far away. This suggests that the global inhibitor induced by an extending pseudopod is rapidly degraded upon termination of the pseudopod and possibly replaced by a global activator.

In wild type cells most pseudopodia are formed nearby, by splitting of an existing pseudopod, and occasionally de novo at a longer distance. We observed that a split pseudopod or a new de novo pseudopod have the same probability to become the parental pseudopod for subsequent pseudopod splitting (data not shown). This suggests that the present pseudopod, formed either de novo or by splitting, produces a local activator that increases the probability to initiate a new pseudopod within the area of the present pseudopod. This local activator may act in conjunction with an inhibitor that represses pseudopod formation in other parts of the cell. A product of the PLA2 pathway could be this local activator since deletion of PLA2 inhibits splitting. We have no molecular model for this observation, mainly because it is unknown how PLA2 affects cell behavior: the substrate of PLA2 is unknown, and it is unclear whether the fatty acid, the lyso-phospholipid, or their metabolites affects pseudopod formation. Of possible importance is the observation that the emerging pseudopod grows significantly longer in pla2-null cells than in wild type cells (13 s in wild type versus 27 s in pla2-null cells, see table S1). The extension of de novo pseudopodia is enhanced in cells lacking guanylyl cyclase, in agreement with previous observations demonstrating that a cGMP pathway induces myosin filaments in the rear of the cell, resulting in the inhibition of pseudopodia [19]. Since cGMP diffuses rapidly whereas lipids diffuse slowly, we suggest that the formation of split versus de novo pseudopodia is regulated by the combination of a local activator (product of PLA2) and a global inhibitor (cGMP) of pseudopod formation.

The probability where to split a pseudopod is distributed unevenly along the boundary of the present pseudopod. The projection map reveals that the probability to extend a new pseudopod at the front ∼1 µm is ∼3-fold lower than at the remaining part of the pseudopod. We realized that it may be difficult to recognize two successive pseudopodia that are extended in the same direction. The computer algorithm has a special subroutine to discriminate between one long-lived pseudopod and two normal pseudopodia extended in a similar direction [12]. Furthermore, visual inspection, although may suffer the same caveat, does provide evidence for wrong interpretation of pseudopodia at the tip. Finally, the growth period of pseudopodia that emerge within 1 µm from the tip is not different from that of other pseudopodia, suggesting that we did not underestimate the number of pseudopodia formed at the tip by merging two normal pseudopodia in one long-lived pseudopod.

Since pseudopod splitting at the tip is inhibited, most new pseudopodia emerge at the side of the present pseudopod. Pseudopod splitting occurs ∼3-fold more frequently alternating right/left than consecutive right/right or left/left. This suggest that the top ∼1 µm and one side of the pseudopod have a ∼3-fold lower probability to extend a new splitting pseudopod than the other side of the pseudopod. The inhibited side is the left side of a left directed pseudopod and the right side of a right directed pseudopod. The molecular basis of this regulation is presently unknown; we have not identified a mutant with altered right/left bias of one-way splits. However, a deletion mutant of PIR121 has been characterized with extensive Y-splitting, suggesting high pseudopod inducing activity at both sides of the pseudopod just after the tip [21]. PIR121 is a component of the SCAR complex that activates Arp2/3-dependent actin nucleation.

In summary, the projection map suggests that the induction of a new pseudopod is regulated by an inhibitor in the cell body (cGMP), by a general activator in the pseudopod (PLA2 product), by a local inhibitor at the tip of the pseudopod, and by a transversal activator in the pseudopod to induce the right/left bias. The probability frequency distributions that were obtained in the present study for the time and position of pseudopod initiation can be used to verify stochastic models with local and global inhibitors and activators of pseudopod formation. In addition, further experiments to determine split/de novo and right/left bias are required, not only in signaling mutants, but also in mutants with modified cytoskeleton, such as the Arp2/3 complex, formins and associated proteins, since these components are critical for the initiation of actin filaments [22], [23].

In this study we have addressed cell movement from the perspective of the pseudopod. The results show that pseudopod extension in the absence of external cues is not random but highly co-coordinated, which may form the basis for internal and external cues to direct cell movement. Our observations raise numerous interesting questions: Do internal cues, such as starvation, affect pseudopod extension? Is cell movement of starved cells more persistent because cells extend pseudopodia more frequently by splitting than de novo [10]? Does the position of the nucleus or the microtubule-organizing center play a role in the position of pseudopod extension [24]–[26]? Furthermore, how do external cues, such as chemoattractants, make use of this co-coordinated pseudopod extension to bias cell movement in the direction of a gradient of chemoattractant [2]? Do chemoattractants induce a bias of the direction of pseudopod extension, or a bias of the position where the pseudopod emerges? Are pseudopodia extended still perpendicular to the surface as in buffer, or are they bent towards the gradient of attractant? How are pseudopodia extended by mutants with defects in pathways that are known to do be involved in chemotaxis, such as cGMP, PI3K, TOR/PKB and PLA2 [16], [27]–[29]? The pseudopod analysis tool may help to answer these eminent questions on the regulation of cell movement by internal and external cues.

## Methods

The strains used are wild type AX3, *pi3k*-null strain GMP1 with a deletion of *pi3k1* and *pi3k2* genes [30], *pla2*-null with a deletion of the *plaA* gene [31], *sgc/gca*-null cells (abbreviated as *gc*-null cells) with a deletion of *gca* and *sgc* genes [32], and *sgc/pla2*-null cells with a deletion of *sgc* and *pla2A* genes [16]. Cells were grown in HG5 medium (contains per liter: 14.3 g oxoid peptone, 7.15 g bacto yeast extract, 1.36 g Na_2_HPO_4_·12H_2_O, 0.49 g KH_2_PO_4_, 10.0 g glucose), harvested in PB (10 mM KH2PO4/Na2HPO4, pH 6.5), and allowed to develop in 1 ml PB in a coated 6-wells plate (Nunc). Movies were recorded with an inverted light microscope (Olympus Type CK40 with a LWD A240 20× numerical aperture 0.4 objective) fitted with a JVC TK-C1381 CCD camera. Digital images were captured at a rate of 1 frame/s on a PC using VirtualDub software and Indeo video 5.10 compression. The field of observation was 358×269 µm

### Semi-automatic pseudopod tracking

Images were analyzed using ImageJ (http://rsb.info.nih.gov/ij/) with a custom made macro that provides a semi-automatic method to characterize pseudopodia. The investigator identifies the start and final position of a pseudopod growth. The macro exports the frame number and x,y-coordinates of these positions, and prints a hard-copy arrow on the relevant frames of the movie.

### Fully automatic pseudopod tracking

The automated pseudopod tracking algorithm Quimp3 [12] is a macro for the open source program ImageJ (http://rsb.info.nih.gov/ij/) and is written as an extension of the Quimp2 program [14]. The package can be downloaded from the site that also contains the previous versions of Quimp: http://www2.warwick.ac.uk/fac/sci/systemsbiology/staff/bretschneider/quimp. A detailed description of Quimp3 is presented in [12] and in the help file of the package.

The phase contrast movie was converted to a black and white movie using the “phase contrast to BW” macro that is included in the Quimp3 package. Some manual adjustment was required to close a few gaps in the cell silhouette. The resulting file was used as input file for the Quimp3 analysis. The pseudopodia were detected using the default parameters of the macro. The Quimp3 produces a data result containing quantitative data for each pseudopod such as the x,y,t coordinates at start and end of the growing phase, the surface area (µm^2^), area change (µm^2^/s), and qualitative data such as the assignment of split versus de novo. The pseudopodia can be drawn on top of the contours of the cell, using colour codes for the different pseudopod types (see figure 1B).

### Data analysis

The result tables of the manual and automatic pseudopod tracking were analyzed using Excel. Primary calculations are size, extension period, and direction of each individual pseudopod. Secondary calculations were made on the connection between subsequent pseudopodia, and include time period between pseudopodia, angle between present and previous pseudopod(s), and distance between start of present pseudopod and end of previous pseudopod.

Data collection and analysis assumes two-dimensional cells and pseudopodia, which is obviously incorrect. Cells move on a 2D agar surface, which implies that the movement in the plane of the agar surface is more important for understanding cell translocation that movement of the pseudopod in the z-direction. In addition it is extremely difficult, if not impossible, to obtain 3D information on pseudopod extension with a 1 s time resolution, and we suspect it will be difficult to extract and analyze pseudopod vectors in 3D. We are aware that data are obtained and discussed in 2D, and their relevance in 3D should be evaluated; for instance we discuss pseudopod length, which is similar in 2D and 3D, but do not discuss on pseudopod area, because this has a completely different meanings in 2D and 3D.

To select cells for pseudopod analysis, we first determined the displacement during 15 min of all ∼20–30 cells in the field of observation, and then selected the 3–5 cells that have a displacement closest to the mean displacement. A typical database contains information on 200–300 pseudopodia obtained from 6–10 cells from two independent movies. For wild type cells we collected data on 323 pseudopodia as control experiments for the mutants (see table S1) and enlarged the data set for more detailed analysis to in total 724 pseudopodia from 26 cells. For subsets of pseudopodia (e.g. split and de novo pseudopodia) the data for some pseudopodia had to be deleted; e.g. for the analysis of split and de novo pseudopodia, the first pseudopod extended by the cell can not be assigned as split or de novo because it has no parental pseudopod (26), y-splits were not included in the data set (36), and visual inspection suggested that the assignment of 20 pseudopodia by the computer algorithm was ambiguous, yielding 530 split and 112 de novo pseudopodia. The data are presented as the means and standard deviation (SD), or standard error of the means (SEM) where n represents the number of pseudopodia or number of cells analyzed. The statistical significance was tested with paired Students t-test.

## Supporting Information

Table S1 Pseudopod properties of Dictyostelium mutants in buffer
(0.04 MB DOC)

Table S2 Properties of maintained split an de novo pseudopodia
(0.03 MB DOC)

Figure S1 Frequency distribution of the size of split and de novo pseudopodia. The insert shows the mean size with SD (downwards) and SEM (upwards). Data are from 530 split and 112 de novo pseudopodia.
(0.51 MB TIF)

Figure S2 Distance dependency of pseudopod extension and Right/Left bias of split and de novo pseudopodia. A. Frequency distribution of the distance between start of next pseudopod and tip of present pseudopod. The inset shows the means and SD for 530 split and 112 de novo pseudopodia. B. The direction of each pseudopod in the binned interval was assigned as right or left tot the previous pseudopod, which was also assigned right or left to its previous pseudopod. Presented is the ratio of alternating (RL+LR) versus consecutive (RR+LL) pseudopodia. De novo pseudopodia have no R/L bias (also those extended at a relatively short distance), whereas split pseudopodia exhibit a R/L bias that becomes stronger at a longer distance from the tip.
(1.00 MB TIF)

Movie S1 (3.93 MB MOV)
