# Supplementary material for: The Ordered Extension of Pseudopodia by Amoeboid Cells in the Absence of External Cues
Source: PLoS One. 2009 Apr 22;4(4):e5253. doi: 10.1371/journal.pone.0005253 (PMC2668753; doi:10.1371/journal.pone.0005253)
Supplement: Table S1 — Pseudopod properties of Dictyostelium mutants in buffer (0.04 MB DOC) [file pone.0005253.s001.doc]

**Table S1 Pseudopod properties of *Dictyostelium* mutants in buffer**

| Strain | n | Pseudopod size  (μm) | | Pseudopod growth time  (s) | | Pseudopod growth speed  (μm/min) | | Pseudopod interval  (s) | | Cell speed  (μm/min) | | Pseudopod activity  (μm/min) | | Frequency  Split  Pseudopodia  (1/s) | | Frequency  *de novo*  pseudopodia (1/s) | | Persistence  (*a*, # split pseudopodia) | |
| --- | --- | --- | --- | --- | --- | --- | --- | --- | --- | --- | --- | --- | --- | --- | --- | --- | --- | --- | --- |
|  |  | mean | SEM | mean | SEM | mean | SEM | mean | SEM | mean | SEM | Mean | SEM | mean | SEM | mean | SEM | mean | SEM |
| Wild type | 12/323 | 5.24 | 0.17 | 12.95 | 0.31 | 0.49 | 0.02 | 16.01 | 0.17 | 8.53 | 0.64 | 19.63 | 2.05 | 3.37 | 0.48 | 0.56 | 0.10 | 6.00 | 0.58 |
| pi3k-null | 8/216 | 4.54 | 0.40 | 9.63 | 0.56 | 0.55 | 0.04 | 14.43 | 1.27 | 7.82 | 0.49 | 18.88 | 3.12 | 3.95 | 0.30 | 0.73 | 0.11 | 5.86 | 0.56 |
| gc-null | 7/312 | 4.60 | 0.40 | 9.42 | 0.37 | 0.52 | 0.05 | 7.88 | 0.47 | 8.53 | 0.49 | 35.11 | 5.81 | 3.52 | 0.33 | 2.35 | 0.25 | 2.50 | 0.20 |
| pla2-null | 8/208 | 7.73 | 0.49 | 27.08 | 3.12 | 0.35 | 0.05 | 32.88 | 3.90 | 7.52 | 0.73 | 16.75 | 1.67 | 1.51 | 0.18 | 0.68 | 0.06 | 2.43 | 0.38 |
| sgc/pla2-null | 8/219 | 5.33 | 0.75 | 19.21 | 4.28 | 0.33 | 0.03 | 18.36 | 2.92 | 6.03 | 0.30 | 18.01 | 1.71 | 1.61 | 0.27 | 2.30 | 0.27 | 0.70 | 0.12 |

Cells have been starved for 5 hours; n is the number of experiments; two values are given, the number of cells and the number of pseudopodia, respectively. Data were obtained in parallel from two movies for each strain, and are presented as the means and standard error of the means where n represents the number of cells analyzed. Cell speed is the instantaneous speed measured at 8s per frame. Pseudopod activity is the total size of pseudopodia extended per minute, and given by 60*(pseudopod size)/(pseudopod interval). Persistence is given as the number of split pseudopodia in between two de novo pseudopodia.
