# Supplementary material for: The Ordered Extension of Pseudopodia by Amoeboid Cells in the Absence of External Cues
Source: PLoS One. 2009 Apr 22;4(4):e5253. doi: 10.1371/journal.pone.0005253 (PMC2668753; doi:10.1371/journal.pone.0005253)
Supplement: Table S2 — Properties of maintained split an de novo pseudopodia (0.03 MB DOC) [file pone.0005253.s002.doc]

**Table S2. Properties of maintained split an *de novo* pseudopodia**

| Property | split | *de novo* |
| --- | --- | --- |
| extension period (sec) | 12.4 +/- 4.1 (346) | 12.7 +/- 6.7 (94) |
| pseudopod size (μm) | 5.5 +/- 1.9 (396) | 5.1 +/- 2.2 (94) |
| pseudopod interval (sec) |  | |
| split-split | 30.0 +/- 9.5 (342) | |
| split-*de novo* | 28.6 +/- 8.9 (83) | |
| *de novo*-split | 27.0 +/- 7.2 (79) | |
| *de novo*-*de novo* | 30.7 +/- 9.5 (21) | |
| all | 29.4 +/- 8.9 (525) | |

The data shown are the means and standard deviation with the number of observations in parenthesis; these numbers represent the relative occurrence of the events, except for *de novo*-*de novo* which is very rare in wild type and has been searched for. In *sgc/pla2*-null cells *de novo*-*de novo* occurs more frequent and occurs at an interval of 34 +/- 9 (n=68).
