# Supplementary figures and images for: The Ordered Extension of Pseudopodia by Amoeboid Cells in the Absence of External Cues
Source: PLoS One. 2009 Apr 22;4(4):e5253. doi: 10.1371/journal.pone.0005253 (PMC2668753; doi:10.1371/journal.pone.0005253)

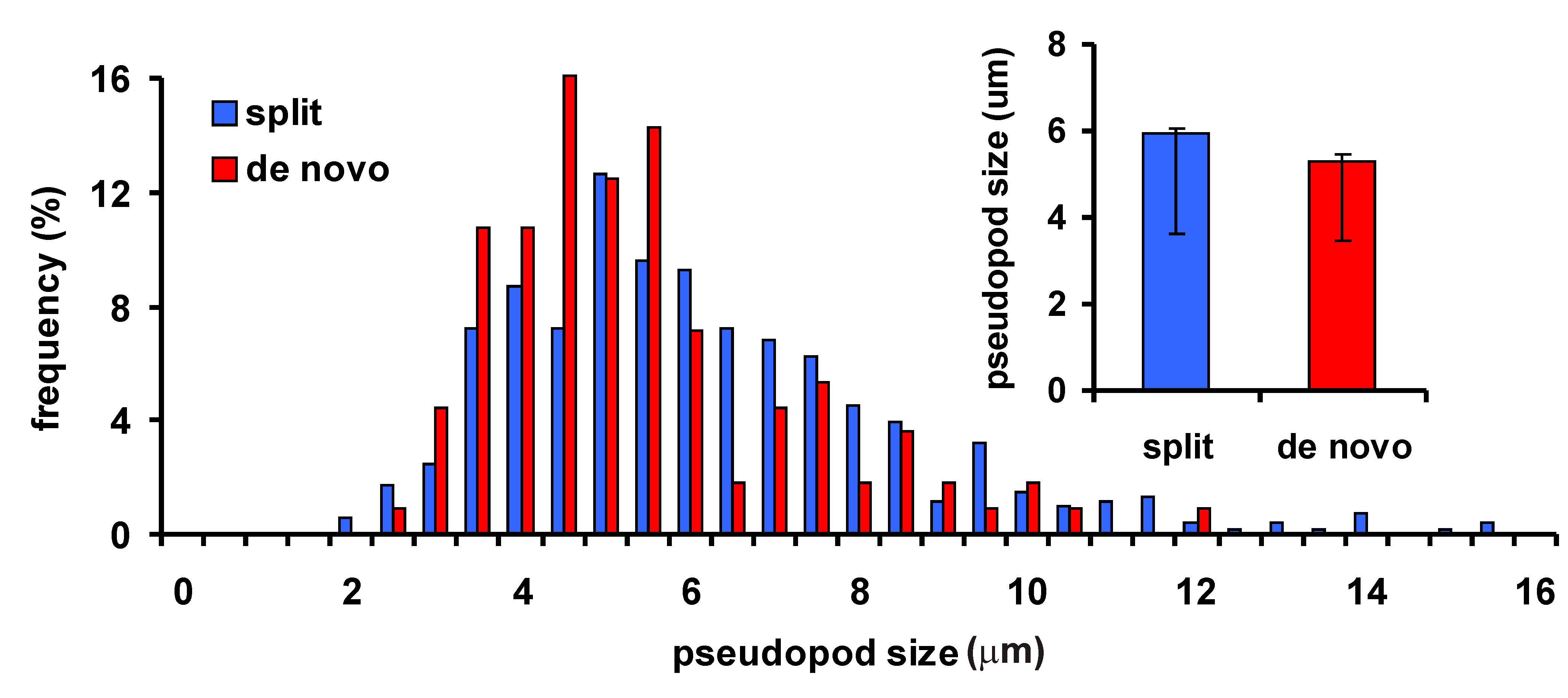

Supplement: Figure S1 — Frequency distribution of the size of split and de novo pseudopodia. The insert shows the mean size with SD (downwards) and SEM (upwards). Data are from 530 split and 112 de novo pseudopodia. (0.51 MB TIF) [file pone.0005253.s003.tif]

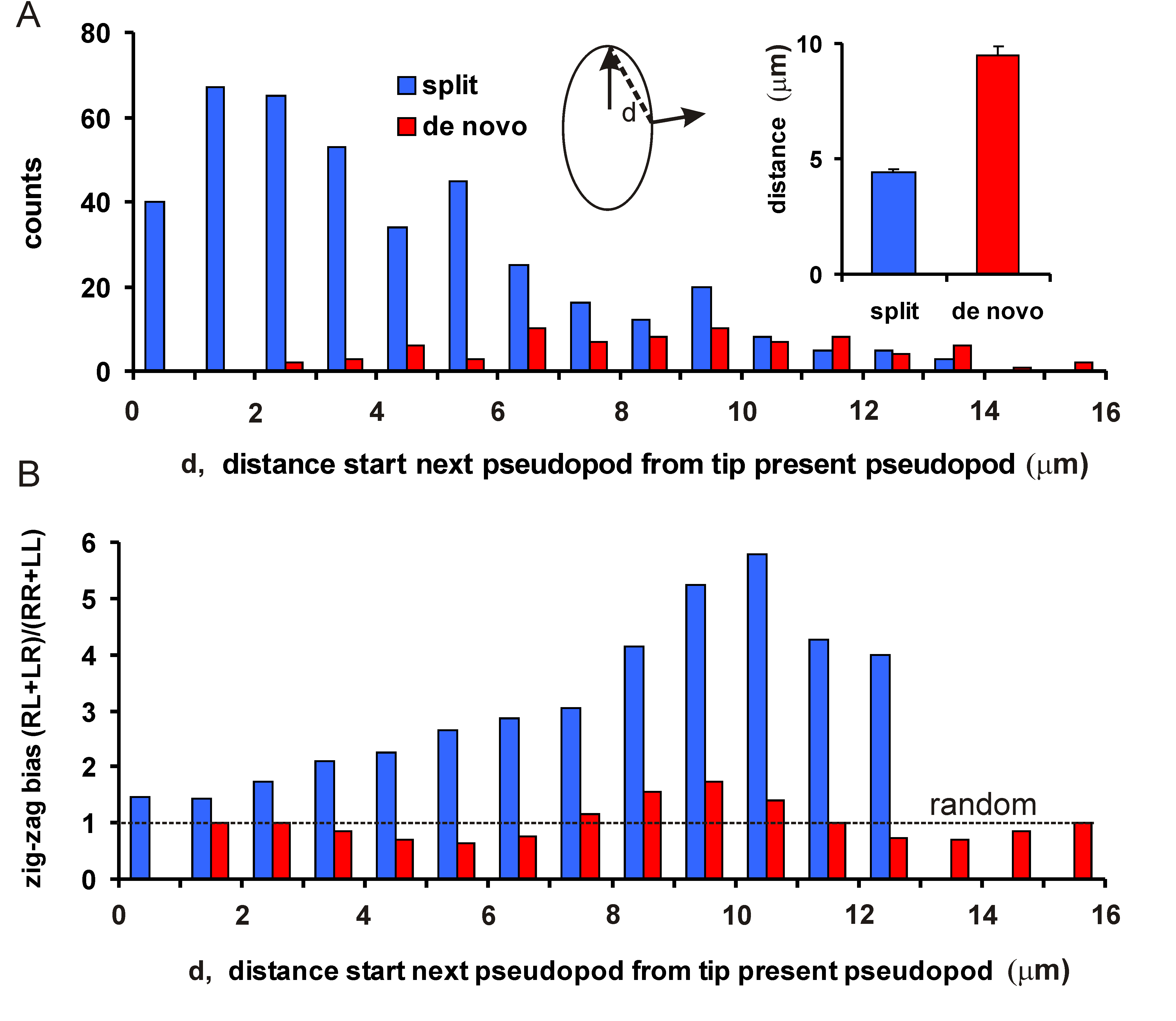

Supplement: Figure S2 — Distance dependency of pseudopod extension and Right/Left bias of split and de novo pseudopodia. A. Frequency distribution of the distance between start of next pseudopod and tip of present pseudopod. The inset shows the means and SD for 530 split and 112 de novo pseudopodia. B. The direction of each pseudopod in the binned interval was assigned as right or left tot the previous pseudopod, which was also assigned right or left to its previous pseudopod. Presented is the ratio of alternating (RL+LR) versus consecutive (RR+LL) pseudopodia. De novo pseudopodia have no R/L bias (also those extended at a relatively short distance), whereas split pseudopodia exhibit a R/L bias that becomes stronger at a longer distance from the tip. (1.00 MB TIF) [file pone.0005253.s004.tif]
